# Editorial: Non-invasive brain stimulation: from basic science to clinical application

**DOI:** 10.3389/fneur.2026.1988104

**Published:** 2026-09-22

**Authors:** Hakjoo Kim, Priyam Rastogi, Kevin A. Caulfield, Simone Russo

**Affiliations:** 1 Brain Stimulation Mechanisms Lab, Division of Depression and Anxiety Disorders, McLean Hospital, Belmont, MA, United States; 2 Department of Psychiatry, Harvard Medical School, Boston, MA, United States; 3 Department of Electrical and Computer Engineering, Iowa State University, Ames, IA, United States; 4 Brain Stimulation Division, Department of Psychiatry and Behavioral Sciences, College of Medicine, Medical University of South Carolina, Charleston, SC, United States; 5 Wallace H. Coulter Department of Biomedical Engineering, Georgia Institute of Technology and Emory University, Atlanta, GA, United States

**Keywords:** neuroplasticity, non-invasive brain stimulation, transcranial direct current stimulation, transcranial electrical stimulation, transcranial magnetic stimulation, transcranial ultrasound stimulation

Non-invasive brain stimulation (NIBS) techniques are used both as causal probes of brain function and as clinically established or investigational interventions for neurological and psychiatric disorders. Transcranial electrical stimulation (tES), transcranial magnetic stimulation (TMS), and transcranial ultrasound stimulation (TUS) differ in their physical principles, spatial resolution, and biological effects, yet share a central translational challenge: stimulation must engage the right neural system, in the right patient, at an appropriate dose, and ultimately produce clinically meaningful benefit. This may explain why compelling neurophysiological effects do not always yield consistent therapeutic benefit (1, 2). This Research Topic was convened to bridge basic mechanisms and clinical applications. Liu et al. map a broader movement-disorder electrical-stimulation literature dominated by deep brain stimulation and Parkinson's disease, while identifying growing interest in neuroplasticity, connectomics, adaptive stimulation, and non-invasive modalities. Collectively, the contributions show that progress depends less on any single modality than on aligning target selection, target-engagement measures, intervention design, patient phenotype, and clinical endpoint.

The first question is where to stimulate. Historically, NIBS has targeted superficial cortex to indirectly influence deeper regions through connected circuitry. Copersino et al. extend this perspective by reframing TMS as a probe of network dynamics in co-occurring alcohol use disorder and major depression, using the superficially accessible temporoparietal junction as an entry point to the ventral attention network. At a more local circuit scale, using dual-coil paired-pulse TMS, Kim et al. compare effective connectivity to the primary motor cortex (M1) across conditioning sites 4, 5, 6, or 7 cm anterior to Cz, observing M1 facilitation only at 4 cm and highlighting the spatial specificity of scalp-based targeting. Beyond superficial targets, Ivanov et al. provide a scoping review of temporal interference stimulation as a potential approach for non-invasive deep-brain modulation, emphasizing unresolved mechanisms, modeling assumptions, sham design, and long-term safety. For amyotrophic lateral sclerosis (ALS), Keihani et al. propose a novel translational TUS-TMS framework leveraging the spatial precision of focused ultrasound to interrogate motor-cortical hyperexcitability. In sum, emerging and established NIBS techniques may target different nodes within a given network, and further work toward identifying optimal strategies is warranted (3).

The second question is whether the intended circuit was engaged. Neuroimaging and electrophysiology can guide stimulation and provide neural readouts, creating a foundation for brain-state-dependent and eventually closed-loop interventions (4). Keihani et al. synthesize literature on TMS-electromyography and TMS-electroencephalography markers of upper motor neuron dysfunction in ALS. In stroke rehabilitation, the systematic review by Zou et al. coalesces early evidence supporting the safety and feasibility of combining TMS and functional near-infrared spectroscopy, with several studies relating hemodynamic changes to motor improvement. However, biomarker-guided closed-loop treatment remains an objective rather than an established practice due to small samples, heterogeneous protocols, and the predominance of measurements obtained before and after stimulation rather than in real time. Future research may move toward increasingly personalized, real-time, and closed-loop approaches to identify and engage the optimal circuit for each individual.

The third question concerns dose and intervention design. Dose is multidimensional and nonlinear: magnitude, temporal structure, and spatial configuration define the *delivered* dose, while anatomy shapes the *received* dose, and brain state influences response (5). Zhang et al. report inconsistent effects of transcranial direct current stimulation (tDCS) on muscle strength in healthy and athletic populations and highlight protocol and individual variability. In post-stroke rehabilitation, Xu L. et al. report greater motor and functional gains with combined central and peripheral magnetic stimulation than with either approach alone. For proprioception, peripheral stimulation alone and the combined protocol both outperform central stimulation alone. Zhao et al. provide exploratory evidence that high-dose accelerated intermittent theta-burst stimulation over M1 may improve gait and selected clinical outcomes in cerebral small vessel disease. Combinatorial strategies are further examined by Wu et al., whose study suggests that multi-target repetitive TMS (rTMS) added to acupuncture may improve central pain and depressive symptoms, without establishing an independent or synergistic rTMS effect. The meta-analysis by Zhou et al. shows no significant analgesic effect of tDCS alone for chronic nonspecific low back pain, whereas tDCS combined with active interventions suggests a significant analgesic effect, but the certainty of evidence is very low. Together, these studies argue against a universal “more stimulation is better” model and favor indication- and context-specific optimization.

The need for such optimization is reflected in uneven clinical translation across disorders and modalities. Small samples, methodological heterogeneity, protocol variability, and differing outcome measures limit cross-study comparisons. In post-stroke pain, Cox et al. review invasive and non-invasive approaches, noting promising signals but uncertain comparative efficacy and durability. For neuropathic pain after spinal cord injury, Luo et al. report a favorable pooled rTMS effect, but with substantial heterogeneity and low-to-moderate certainty. Xu H. et al. describe a randomized, double-blind, placebo-controlled protocol designed to test adjunctive rTMS in postherpetic neuralgia with depression; no efficacy conclusion can yet be drawn. In treatment-resistant schizophrenia, Alhassan et al. found no significant pooled effects on global symptoms, negative symptoms, or auditory hallucinations. Although signals emerged in exploratory subgroups involving prefrontal stimulation and patients with prominent negative symptoms, the small number of studies and absence of formal subgroup-difference tests preclude firm conclusions. These contrasting findings reinforce that a modality label alone does not predict clinical efficacy; disorder-specific circuits, protocols, co-interventions, and patient selection matter.

In summary, NIBS represents an ever-developing field where the path forward is precision with rigor. Safety must be considered in relation to modality, dose, population, and context, guided by established TMS recommendations (6), updated tES guidance (7), and standardized TUS reporting (8) and methodology (9). Future studies should prioritize biomarker-guided intervention selection and target-engagement verification, report delivered and estimated received doses, use credible sham and blinding, prespecify meaningful outcomes, assess durability, and personalize stimulation using physiological or imaging markers. Pharmacological combinations and concurrent functional magnetic resonance imaging, both within the intended scope of this Research Topic, remain underrepresented and warrant systematic investigation. By linking mechanism, measurement, intervention design, and clinical testing, this Research Topic defines a translational roadmap toward safer, reproducible, and individually optimized NIBS.
